# Factors related to early weaning in babies born at term in a public maternity

**DOI:** 10.1590/2317-1782/20242024030en

**Published:** 2024-08-05

**Authors:** Sandra Raquel de Melo Gomes, Mirelly Sabrina Santos Silva, Andréa Rodrigues Motta, Estevam Barbosa de Las Casas, Renata Maria Moreira Moraes Furlan

**Affiliations:** 1 Programa de Pós-graduação em Ciências Fonoaudiológicas, Faculdade de Medicina, Universidade Federal de Minas Gerais – UFMG - Belo Horizonte (MG), Brasil.; 2 Residência Multiprofissional, Hospital Metropolitano Odilon Behrens - Belo Horizonte (MG), Brasil.; 3 Departamento de Fonoaudiologia, Faculdade de Medicina, Universidade Federal de Minas Gerais – UFMG - Belo Horizonte (MG), Brasil.; 4 Departamento de Engenharia de Estruturas, Escola de Engenharia, Universidade Federal de Minas Gerais – UFMG - Belo Horizonte (MG), Brasil.

**Keywords:** Breast Feeding, Weaning, Child Nutrition, Sociodemographic Factors, Maternal and Child Health

## Abstract

**Purpose:**

to analyze how socioeconomic, pregnancy and childbirth factors relate to the feeding situation in the sixth month of life of full-term babies.

**Methods:**

longitudinal observational study, with 98 mothers of full-term babies. Data collection was structured by capturing information regarding the clinical history and moment of birth in the babies' medical records, followed by the application of two questionnaires to the postpartum women, with questions regarding sociodemographic data, pre- and post-pregnancy data and the baby's nutrition. baby, the first being answered during hospital stay and the second, by telephone, in the 6th month of life. A descriptive analysis of the data was performed, using the frequency distribution of categorical variables, inferential analysis using Pearson's Chi-square test and multivariate analysis using binary logistic regression, adopting, for inclusion in the final model, the significance level of 5%.

**Results:**

there was an association between exclusive breastfeeding in the 6th month and maternal education and between the period of food introduction and family income. Mothers with higher education were 4.82 times more likely to breastfeed their children exclusively until the sixth month. Families with lower income (up to one minimum wage) were 2.54 times more likely to start food introduction before the sixth month than families with higher income.

**Conclusion:**

higher maternal education was a predictive factor for exclusive breastfeeding at the 6th month and higher military income was a predictive factor for introducing food after the 6th month.

## INTRODUCTION

Breast milk is the most appropriate food for baby nutrition. The World Health Organization (WHO)^(1)^ and the Brazilian Ministry of Health^(2)^ recommend that newborns receive breast milk as early as the first hour of life and maintain it exclusively until the 6^th^ month. After this period, they recommend maintaining breastfeeding (BF) in addition to other foods until the children are at least 2 years old.

The Brazilian 2019 National Child Food and Nutrition Study^(3)^ investigated BF prevalence and practices in Brazilian children under 2 years old and pointed out that it lasts on average less than the recommended 6 months for exclusive BF (EBF) and 2 years or more for supplemented BF. The average was found to be 15.9 months for BF and just 3 months for EBF – only 45.8% of children up to 6 months old received EBF. This scenario is far from the target established by the WHO: at least 70% of children under 6 months old on EBF by 2030^(3)^.

BF benefits the babies’ health as a source of nutrients and antibodies, helps reduce infant mortality, strengthens the bond between mother and child, stimulates oral-motor development, contributes to the development of digestive, cutaneous, and respiratory microbiota, and has a positive influence on the children’s health throughout their lives^(2,4)^. As for the women's health, BF prevents breast, ovarian, and uterine cancer, reduces the risk of developing type 2 diabetes, and contributes to mental health^(2)^.

Despite all these benefits, the decision to breastfeed is not immediate and simple. Depending on the context where the woman lives, BF can pose difficulties and insecurities influenced by cultural and emotional burdens. During pregnancy and postpartum, they are exposed to many opinions, beliefs, and reports of experiences from people in their life cycle, which may or may not encourage BF^(5)^. Beliefs and myths related to BF, breast milk, and the onset of nipple pain and trauma – often due to a lack of maternal experience and support and guidance from health professionals – can motivate early weaning^(5)^.

Such difficulties lead mothers to supplement or replace breast milk with infant formulas and other foods, which can result in the baby's weaning^(6)^. Serving formula in a bottle can change the baby's sucking pattern, resulting in greater difficulty in sucking at the mother's breast and, in turn, leading to refusal of the breast and reduced breast milk production due to lack of stimulation^(7)^. Serving other foods early exposes the child to the risk of colic and/or diarrhea, as the baby's body is not yet prepared to process these substances before 6 months^(7)^. As they receive other foods, they feed less often, which further decreases stimulation and breast milk production^(7)^.

Thus, knowing the benefits that breast milk brings, it is important to know the factors that influence BF continuation to understand and create ways to support mothers in this process, avoiding the losses related to early weaning. Hence, this study aimed to analyze how socioeconomic, pregnancy, and childbirth factors relate to the feeding status in the 6^th^ month of life of full-term babies.

## METHOD

This is a longitudinal observational study with a non-randomized sample of mothers of full-term newborns hospitalized in the rooming-in ward of the Odilon Behrens Hospital. The study was approved by the institution's Research Ethics Committee under evaluation report number 4.480.984. All participants signed an informed consent form.

The inclusion criteria were mothers over 18 years old, full-term newborns (gestational age greater than or equal to 37 weeks), breastfeeding, and staying in the rooming-in ward.

The exclusion criteria were as follows: mothers whose newborns had severe congenital heart or lung diseases, genetic syndromes, or orofacial structural changes (as these clinical conditions can interfere with BF, as they pose the newborn to greater risks of sucking/swallowing/breathing incoordination), mothers with diseases or therapeutic procedures that contraindicated BF or that could interfere with it, and mothers who did not answer the second research questionnaire.

Data were collected by surveying information on clinical history and birth from the babies' medical records, followed by administering two questionnaires to the participants, one during hospital stay and the other 6 months later (Appendices 1 and 2).

The first questionnaire had two parts and was applied during hospital stay (postpartum) in January and February 2020. The first part of this questionnaire addressed sociodemographic data (maternal age, color/race, marital status, education, profession, and family income) and data from previous pregnancies (number of children). Its second part investigated data on the current pregnancy and BF (number of prenatal consultations, sex of the baby, type of delivery, EBF at hospital discharge and BF complaints).

The second questionnaire was administered 6 months after the child's birth, via phone call. The questionnaire had the following questions about the baby's current feeding situation: “Was the baby on EBF until the 6th month?” – answer options: yes or no; “What was the BF type in the 6th month?” – answer options: EBF, supplemented BF, mixed or partial BF, and formula (Chart 1)^(8)^; “Are you breastfeeding?” answer options: yes or no; “Did you start offering baby food?” – answer options: yes or no; and “When did you start serving baby food?” – the participants answered this question freely; the researchers categorized the answers into “up to the 4^th^ month” and “from the 5^th^ month” for statistical data analysis. This variable also had the “not applicable” category for babies who were not yet eating food at the time of the phone call.

**Chart 1:** Classification of the types of breastfeeding

Breastfeeding type	Definition
Exclusive breastfeeding	When the child received only breast milk.
Supplemented breastfeeding	When the child received, in addition to breast milk, any solid or semi-solid food to complement but not replace it.
Mixed or partial breastfeeding	When the child received breast milk and other types of milk.
Infant formula	When the child received only types of milk other than breast milk.

Source: Brasil^(8)^

Data from the two questionnaires were recorded in a Microsoft Excel spreadsheet and then subjected to inferential statistical analysis. The response variables of this research were the presence of EBF until the 6^th^ month, the presence of some BF type at the 6^th^ month, the BF type practiced in the 6^th^ month, and the age at which food was introduced. The following explanatory variables were evaluated: maternal age, marital status, education, color/race, profession, family income, number of children, type of birth, prenatal care, sex of the baby, EBF at hospital discharge, and BF complaints.

A descriptive analysis of the data was performed with the frequency distribution of categorical variables. Pearson's chi-square test was used for association analyses, considering statistically significant associations those with a p-value ≤ 0.05. Pearson's chi-square test was also used for a pairwise analysis between maternal education categories and EBF up to the 6^th^ month; those with a p-value ≤ 0.05 were considered as statistically significant associations. SPSS software, version 25.0, was used to enter, process, and analyze data.

Multivariate analysis was also performed with binary logistic regression. The explanatory variables selected for the model were those associated in bivariate analyses with the presence of BF at the 6^th^ month, presence of EBF at the 6^th^ month, and introducing food before the 6^th^ month, setting the sinificance level at 20% (p =< 0.20). For inclusion in the final model, the significance level was set at 5%. The magnitude of the associations was assessed with odds ratios and their respective 95% confidence intervals.

## RESULTS

In the first stage, 224 participants answered the questionnaire. In the second stage, 126 participants were not located and were excluded from the study. Thus, 98 participants made up the sample for this research.

Most mothers who participated in the study were 21 to 35 years old (75.5%); 64 mothers were single (65.3%); the majority had graduated from high school (73.5%) while the remainder were equally divided between middle school and higher education. Most participants classified themselves as multiracial (63.3%), followed by Black, White, and East Asian, with the lowest percentage. Most mothers were self-employed or worked at home and reported a family income of two or more minimum wages (55.1%).

Most participants (83.7%) reported having six or more prenatal consultations, and the majority had natural births (55.1%) and were multiparous (53.1%). As for the babies, 56.1% were males, and the majority (89.8%) were on EBF at the time of postpartum hospital discharge.

Also, 60.2% of participants had BF complaints, mostly sore or cracked nipples and BF pain. Most babies (83.7%) did not receive EBF until the 6^th^ month of life, and 64.3% of babies had no contact with breast milk in the 6^th^ month. The predominant BF type in the 6th month of life was formula (34.7%), followed by mixed (31.6%), supplemented (17.3%), and EBF (16.3%). Most babies (41.8%) had food introduced after the 5^th^ month, 17.3% started at 4 months or earlier, and 40.8% had not started.

The association analysis between EBF until the 6^th^ month of life and sociodemographic data and data from previous pregnancies and the current pregnancy (Table 1) demonstrated a statistically significant association between EBF in the 6th month of life and maternal education. The pairwise analysis between education categories showed a statistical difference between higher education and high school (p = 0.010). This analysis demonstrated that mothers graduated from high school were less prone to EBF than those with higher education. No statistically significant association was identified in the analysis between nonexclusive BF at the 6^th^ month and sociodemographic and pre/postnatal data, also described in Table 1.

**Table 1:** Association analysis between breastfeeding up to the sixth month, exclusive breastfeeding up to the sixth month, and sociodemographic and pre/post-natal data

**Variables**	**Exclusive breastfeeding up to the 6^th^ month**			**Breastfeeding at the 6^th^ month**		
	**Yes N (%)**	***No N (%)***	**p-value**	**Yes N (%)**	**No N (%)**	**p-value**
**Maternal age**						
Up to 20 years	2 (12.5)	12 (14.6)	0.931	2 (10.5)	10 (15.9)	0.595
21 to 35 years	12 (75.0)	62 (75.6)		16 (84.2)	46 (73.0)	
Above 35 years	2 (12.5)	8 (9.8)		1 (5.3)	7 (11.1)	
**Marital status**						
Single	10 (62.5)	54 (65.9)	0.797	11 (57.9)	43 (68.3)	0.404
Married	6 (37.5)	28 (34.1)		8 (42.1)	20 (31.7)	
**Maternal education**						
Middle school ^A,B^	3 (18.8)	10 (12.2)	0.004*	2 (10.5)	8 (12.7)	0.801
High school ^A^	7 (43.8)	65 (79.3)		16 (84.2)	49 (77.8)	
Higher education ^B^	6 (37.4)	7 (8.5)		1 (5.3)	6 (9.5)	
**Color/race**						
Black	5 (31.3)	17 (20.7)	0.603	1 (5.3)	16 (25.4)	0.293
East Asian	1 (6.2)	2 (2.4)		0 (0.0)	2 (3.2)	
White	2 (12.5)	9 (11.0)		2 (10.5)	7 (11.1)	
Multiracial	8 (50.0)	54 (65.9)		16 (84.2)	18 (60.3)	
**Occupation**						
Self-employed or works at home	9 (56.3)	53 (64.6)	0.525	13(68.4)	40 (63.5)	0.694
Works for an employer	7 (43.7)	29 (35.4)		6 (31.6)	23 (36.5)	
**Family income**						
Up to one minimum wage	6 (37.5)	38 (43.6)	0.515	10 (52.6)	28 (44.4)	0.530
Two or more minimum wages	10 (62.5)	44 (53.7)		9 (47.4)	35 (55.6)	
**Primiparity**						
Primiparous	11 (68.8)	35 (42.7)	0.056	7 (36.8)	28 (44.4)	0.557
Multiparous	5 (31.2)	47 (57.3)		12 (63.2)	35 (55.6)	
**Type of delivery**						
Cesarean	10 (62.5)	34 (41.5)	0.122	10 (52.6)	24 (38.1)	0.260
Natural	6 (37.5)	48 (58.5)		9 (47.4)	39 (61.9)	
**Prenatal care**						
Up to six consultations	2 (12.5)	13 (16.0)	0.720	5 (26.3)	8 (12.9)	0.163
Six or more consultations	14 (87.5)	68 (84.0)		14 (73.7)	54 (87.1)	
**Sex of the baby**						
Females	7 (43.8)	36 (43.9)	0.991	9 (47.4)	27 (42.9)	0.728
Males	9 (53.6)	46 (56.1)		10 (52.6)	36 (57.1)	
**Exclusive breastfeeding at hospital discharge**						
Yes	15 (93.8)	73 (89.0)	0.568	34 (97.1)	54 (85.7)	0.081
No	1 (6.3)	9 (11.0)		1 (2.9)	9 (14.3)	
**Breastfeeding complaints**						
Yes	9 (53.6)	50 (61.0)	0.724	15 (78.9)	35 (55.6)	0.067
No	7 (43.8)	32 (39.0)		4 (21.1)	28 (44.4)	

Pearson’s chi-square test

* = p-value ≤ 0.05

**Caption:** N = number of individuals, varying due to missing data. Different superscript letters indicate statistical differences between groups, whereas the same superscript letters indicate the absence of statistical difference between groups.

The association analysis between the BF type in the 6^th^ month and sociodemographic and pre/postnatal data (Table 2) revealed a lack of statistical significance in any of the associations analyzed.

**Table 2:** Association analysis between the type of breastfeeding and sociodemographic and pre/postnatal data

**Variables**	**Breastfeeding type**					
	**EBF**	**Suppl. BF**	**Mixed BF**	**Formula**	**Total**	**p-value**
	**N (%)**	**N (%)**	**N (%)**	**N (%)**	**N (%)**	
**Maternal age**						
Up to 20 years	2 (14.3)	1 (7.1)	6 (42.9)	5 (35.7)	14 (100.0)	0.881
21 to 35 years	12 (16.2)	15 (20.3)	22 (29.7)	25 (33.8)	74 (100.0)	
Above 35 years	2 (20.0)	1 (10.0)	3 (30.0)	4 (40.0)	10 (100.0)	
**Marital status**						
Single	10 (15.6)	9 (14.1)	21 (32.8)	24 (37.5)	64 (100.0)	0.636
Married	6 (17.6)	8 (23.5)	10 (29.4)	10 (29.4)	34 (100.0)	
**Maternal education**						
Middle school	3 (23.0)	2 (15.4)	2 (15.4)	6 (46.2)	13 (100.0)	0.056
High school	7 (9.7)	14 (19.4)	25 (34.7)	26 (36.2)	72 (100.0)	
Higher education	6 (46.2)	1 (7.7)	4 (30.8)	2 (15.3)	13 (100.0)	
**Color/Race**						
Black	5 (22.7)	1 (4.5)	6 (27.3)	10 (45.5)	22 (100.0)	0.692
East Asian	1 (33.3)	0 (0.0)	1 (33.3)	1 (33.4)	3 (100.0)	
White	2 (18.2)	2 (18.2)	3 (27.3)	4 (36.3)	11 (100.0)	
Multiracial	8 (12.9)	14 (22.6)	21 (33.9)	19 (30.6)	62 (100.0)	
**Occupation**						
Self-employed or works at home	9 (14.5)	12 (19.4)	17 (27.4)	24 (38.7)	62 (100.0)	0.482
Works for an employer	7 (19.4)	5 (13.9)	14 (38.9)	10 (27.8)	36 (100.0)	
**Family income**						
Up to one minimum wage	6 (13.6)	10 (22.7)	12 (27.3)	16 (36.4)	44 (100.0)	0.526
Two or more minimum wages	10 (18.5)	7 (13.0)	19 (35.2)	18 (33.3)	54 (100.0)	
**Primiparity**						
Primiparous	11 (23.9)	6 (13.1)	18 (39.1)	11 (23.9)	46 (100.0)	0.058
Multiparous	5 (9.6)	11 (21.1)	13 (25.0)	23 (44.3)	52 (100.0)	
**Type of delivery**						
Cesarean	10 (22.7)	9 (20.5)	11 (25.0)	14 (31.8)	44 (100.0)	0.290
Natural	6 (11.2)	8 (14.8)	20 (37.0)	20 (37.0)	54 (100.0)	
**Prenatal care**						
Up to six consultations	2 (13.3)	4 (26.7)	3 (20.0)	6 (40.0)	15 (100.0)	0.586
Six or more consultations	14 (17.1)	13 (15.9)	28 (34.1)	27 (32.9)	82 (100.0)	
**Sex of the baby**						
Females	7 (16.3)	8 (18.6)	11 (25.6)	17 (39.5)	43 (100.0)	0.688
Males	9 (16.4)	9 (16.4)	20 (36.3)	17 (30.9)	55 (100.0)	
**Exclusive breastfeeding at hospital discharge**						
Yes	15 (17.0)	17 (19.3)	27 (30.7)	29 (33.0)	88 (100.0)	0.361
No	1 (10.0)	0 (0.0)	4 (40.0)	5 (50.0)	10 (100.0)	
**Breastfeeding complaints**						
Yes	9 (15.3)	14 (23.7)	16 (27.0)	20 (33.3)	59 (100.0)	0.206
No	7 (17.9)	3 (7.7)	15 (38.5)	14 (35.9)	39 (100.0)	

**Caption:** N = number of individuals; EBF = exclusive breastfeeding; Suppl. = supplementary; BF = breastfeeding.

Pearson’s chi-square test.

The association analysis between introducing food before the 6^th^ month and sociodemographic and pre/postnatal data (Table 3) showed a statistically significant association between introducing food and family income. Families with higher income introduced food later than those with lower income. No significant association was found between the most prevalent ages at food introduction (4^th^ and 5^th^ month of the baby's life) and sociodemographic and pre/postnatal data.

**Table 3:** Association analysis between food introduction and sociodemographic and pre/postnatal data

**Variables**	**Food was introduced**			**Age when food was introduced**		
	**Yes N (%)**	**No N (%)**	**p-value**	**Up to the 4^th^ month N (%)**	**From the 5^th^ month N (%)**	**p-value**
**Maternal age**						
Up to 20 years	8 (14.5)	4 (14.8)	0.881	3 (17.6)	6 (14.6)	0.959
21 to 35 years	41 (74.6)	21 (77.8)		12 (70.6)	30 (73.2)	
Above 35 years	6 (10.9)	2 (7.4)		2 (11.8)	5 (12.2)	
**Marital status**						
Single	33 (60.0)	21 (77.8)	0.111	9 (52.9)	27 (65.9)	0.356
Married	22 (40.0)	6 (22.2)		8 (47.1)	14 (34.1)	
**Maternal education**						
Middle school	8 (14.5)	2 (7.4)	0.608	3 (17.6)	5 (12.2)	0.709
High school	42 (76.4)	23 (85.2)		12 (70.6)	33 (80.5)	
Higher education	5 (9.1)	2 (7.4)		2 (11.8)	3 (7.3)	
**Color/race**						
Black	10 (18.2)	7 (25.9)	0.669	4 (23.5)	7 (17.1)	0.218
East Asian	2 (3.6)	0 (0.0)		2 (11.8)	0 (0.0)	
White	6 (10.9)	3 (11.1)		2 (11.8)	4 (9.8)	
Multiracial	37 (67.3)	17 (63.0)		9 (52.9)	30 (73.1)	
**Occupation**						
Self-employed or works at home	38 (69.1)	15 (55.6)	0.228	11 (64.7)	29 (70.7)	0.652
Works for an employer	17 (30.9)	12 (44.4)		6 (35.3)	12 (29.3)	
**Family income**						
Up to one minimum wage	30 (54.5)	8 (29.6)	0.033*	11 (64.7)	20 (48.8)	0.268
Two or more minimum wages	25 (45.5)	19 (70.4)		6 (35.3)	21 (51.2)	
**Primiparity**						
Primiparous	22 (40.0)	13 (48.1)	0.483	6 (35.3)	16 (39.0)	0.790
Multiparous	33 (60.0)	14 (51.9)		11 (64.7)	25 (61.0)	
**Type of delivery**						
Cesarean	25 (45.5)	9 (33.3)	0.295	7 (41.2)	18 (43.9)	0.849
Natural	30 (54.5)	18 (66.7)		10 (58.8)	23 (56.1)	
**Prenatal care**						
Up to six consultations	10 (18.2)	3 (11.5)	0.447	2 (11.8)	9 (22.0)	0.368
Six or more consultations	45 (81.8)	23 (88.5)		15 (88.2)	32 (78.0)	
**Sex of the baby**						
Females	25 (45.5)	9 (33.3)	0.295	4 (23.5)	19 (46.3)	0.106
Males	30 (54.5)	18 (66.7)		13 (76.5)	22 (53.7)	
**Exclusive breastfeeding at hospital discharge**						
Yes	51 (92.7)	22 (81.5)	0.126	16 (94.1)	38 (92.7)	0.844
No	4 (7.3)	5 (18.5)		1 (5.9)	3 (7.3)	
**Breastfeeding complaints**						
Yes	35 (63.6)	15 (55.6)	0.481	11 (64.7)	26 (63.4)	0.926
No	20 (36.4)	12 (44.4)		6 (35.3)	15 (36.6)	

Pearson’s chi-square test

* = p-value ≤ 0.05

**Caption:** N = number of individuals, varying due to missing data and babies included in the “not applicable” category (N = 40) – i.e., those who had not had food introduced by the sixth month

The results of the multivariate analysis (Table 4) indicate that education can be considered a predictive factor for EBF until the 6^th^ month – mothers with higher education were 4.82 times more likely to breastfeed their children exclusively until the 6^th^ month. Family income was a predictive factor for introducing food after 6 months, as families with lower incomes (up to one minimum wage) were 2.54 times more likely to introduce food before the 6^th^ month than those with higher incomes.

**Table 4:** Logistic regression model for exclusive breastfeeding at the sixth month, breastfeeding at the sixth month, and food introduction before the sixth month

Variables	Odds ratio	p-value	95% confidence interval	
			Minimum	Maximum
**EBF in the 6^th^ month**				
Education (higher education)	4.82	0.026	1.209	19.260
Primiparity (primiparous)	1.84	0.345	0.518	6.549
Type of delivery (natural)	0.45	0.191	0.143	1.473
**BF in the 6^th^ month**				
Prenatal care (up to 6 consultations)	2.56	0.154	0.704	9.291
EBF at hospital discharge (yes)	1	-	-	-
Complaints (yes)	2.95	0.081	0.877	9.904
**Food introduction before the 6^th^ month**				
Marital status (married)	1.85	0.177	0.757	4.513
Income (up to one minimum wage)	2.54	0.031	1.091	5.918
EBF at hospital discharge (yes)	2.12	0.276	0.547	8.255

**Caption:** EBF = exclusive breastfeeding, BF = breastfeeding

## DISCUSSION

This study found a significant association between EBF until the 6^th^ month and maternal education. Most mothers in each education category (middle school, high school, and higher education) did not maintain EBF until the 6^th^ month. The majority were high school graduates and did not maintain EBF until the 6^th^ month. On the other hand, the pairwise comparison identified that more mothers with higher education maintained EBF until the 6^th^ month than those who graduated from high school. Thus, the higher education category was included in the logistic regression model, confirming education as a protective factor for EBF until the 6^th^ month, as mothers with higher education were 4.82 times more likely to breastfeed their children exclusively until the 6^th^ month.

These findings are similar to what the literature points out. A literature review by Silva et al.^(6)^ found that lower education is a factor related to early weaning and suggested that the fact that the group of mothers with less education has less access to information explains why they stop breastfeeding early. These data are reaffirmed by Nabate et al.^(9)^, who pointed out that mothers with a lower education level are significantly prone to early weaning. In agreement, Barbosa et al.^(10)^ reported that mothers with less than 8 years of education (incomplete middle school) tend to abandon EBF early and reinforced that women with little or no education are unaware of the importance of EBF for their baby's health. Hence, promoting and expanding educational campaigns for the population could help increase EBF rates.

Other authors^(11)^ also report that low education is associated with shorter BF duration. Based on a study that used data from the 1991, 1997, and 2006 Pernambuco State Health and Nutrition Survey (PESN, in Portuguese), the authors^(11)^ pointed out that women with 9 or more years of education had a higher prevalence of EBF at the 6^th^ month than those with less education, thus configuring higher education as a protective factor for EBF duration. These authors^(11)^ suggest that more prenatal consultations can encourage BF continuation among mothers with lower education, as consultations give an opportunity to provide guidance and strengthen knowledge about BF.

The results of the present study also show a statistically significant association between family income and early food introduction. Most mothers who had not introduced food to their children until the 6^th^ month had a higher family income (two or more minimum wages). The regression model showed that families with lower incomes (up to one minimum wage) were 2.54 times more likely to introduce food before the 6^th^ month than families with higher incomes.

Melo et al.^(12)^ carried out a study with parents of children aged 0 to 2 years from three private schools in Belo Horizonte and Contagem, Brazil, and found that the parents' knowledge about introducing food to children was correlated with aspects of education, occupation outside the home, family income, and having a health insurance. Parents with greater knowledge about introducing food had more education, worked outside the home, and had a higher family income and health insurance.

The literature suggests that the greater the knowledge about complementary feeding, the lower the chance of introducing food early. Furthermore, choosing the child's diet makeup is directly related to the families' purchasing power, which is directly influenced by family income. Thus, child nutrition encompasses sociocultural and economic aspects^(12)^.

Giesta et al.^(13)^ conducted a study with mothers of children aged 4 to 24 months admitted to the pediatric sector or pediatric emergency of a tertiary hospital in Porto Alegre, Brazil. They found a low prevalence of EBF and inadequate food introduction, although most mothers had been guided on complementary feeding by health professionals. Moreover, there was a high prevalence of ultra-processed foods introduced before 6 months of life. These inappropriate practices were more present among older multiparous mothers with lower family income and less education.

The characterization of the sample in the present study – in which most women had a family income of two or more minimum wages, were multiparous, and whose children were receiving infant formula – suggests that these mothers did not introduce food early because they had the financial means to maintain the supply of infant formulas. No need to introduce other foods to meet 100% of the child's needs is identified when infant formula can be maintained.

Understanding the relationship between families’ socioeconomic level, the early introduction of complementary food, and the inadequate supply of food in this phase makes it possible to create health policies that ensure adequate early eating practices that continue throughout childhood, adolescence, and adulthood^(14)^.

Complementary food was introduced to the children in the present study mostly in the 5^th^ month. The remaining children were divided into “up to the 4^th^ month” and “not applicable” (meaning that food had not yet been introduced). The findings of the study by Melo et al.^(12)^ agree with those of the present research, as food was introduced in their sample mostly between 0 and 5 months – which is early, considering the recommendations of the WHO and the Brazilian Ministry of Health. Early food introduction is usually associated with early weaning. Therefore, the factors that influence the decision to stop BF consequently encourage the provision of complementary foods earlier than recommended^(7,15)^.

Early food introduction is common in various developed and developing countries^(16-19)^. A study carried out in the Middle East showed that 78.6% of children in Iraq, 70% of children in the United Arab Emirates, and 52.9% of children in Lebanon receive complementary food between 4 and 6 months old, not following recommendations from the WHO^(18)^. Furthermore, a multicenter study with European countries found that 25% of the children evaluated had started complementary feeding before the 4^th^ month of life, and at least 90% of the children had consumed solid foods by 6 months old^(19)^.

A point that draws attention in the present study regarding the BF type in the 6^th^ month is that most babies were not on either BF or EBF. Of the children evaluated, 34.7% were on infant formula, and the remainder were divided into other categories. Torquato et al.^(20)^ evaluated the BF pattern of children aged 0 to 24 months and found that most children were not breastfeeding. Most of those 6 months or younger were neither on EBF or supplemented BF. Torquato et al.^(20)^ reinforce that the belief that breast milk is insufficient and/or weak is still very strong and deep-rooted, greatly influencing the mothers' decision to serve other types of food (water, juice, other milk, and solid foods) before 6 months. Another notable point is that more than 10% of postpartum women left the hospital not practicing EBF, and only one of them performed EBF at the 6^th^ month. This highlights the importance of actions – e.g., guidance provided by professionals, counseling, and peer support interventions – to initiate and maintain BF immediately after birth^(21)^.

Pinheiro et al.^(16)^, in turn, point out that women decide to stop BF often due to nipple pain and trauma, even though they know the importance and benefits of EBF until the 6^th^ month. Barbosa et al.^(22)^ also identified a high frequency of early weaning in the first months of the baby's life and breast problems as factors associated with discontinuing EBF, observed as early as the maternity ward and persisted. Although the literature considers breast pain and trauma as important aspects for discontinuing BF, and mothers in the present study had BF complaints, these were not associated with early weaning in the sample.

Some limitations should be considered in this study. It had few participants due to the short data collection period, and the collection setting had specific characteristics (a metropolitan hospital that is a reference in the care of high-risk pregnancies). Therefore, the data should not be generalized to other populations. Furthermore, maternal memory can pose a risk of bias in the study, since the second questionnaire had questions that depended on their memory. The strengths of this research include its monitoring with two measurements over time, which minimized biases that would have been present if it had been carried out only in the 6^th^ month. This study is relevant to the literature by elucidating how socioeconomic, pregnancy, and childbirth data are related to babies’ feeding status in the 6^th^ month of life. This understanding can give rise to strategies that help women in BF and introducing food, avoiding early weaning and its consequences.

## CONCLUSION

Maternal education was associated with the presence of EBF in the 6^th^ month – more mothers with higher education provided EBF until the 6^th^ month of life than those graduated from high school. Also, family income was associated with introducing food at the 6^th^ month, as mothers with higher incomes did not introduce complementary food before the 6^th^ month.

## Appendix 1

**Table:** Structured questionnaire applied at the hospital

					DATE:
**PART I – IDENTIFICATION AND SOCIOECONOMIC DATA**					
NAME:					
DATE OF BIRTH:			AGE:		
PLACE OF BIRTH:			MEDICAL RECORD NO.:		
ADDRESS:			PHONE NUMBERS:		
MARITAL STATUS: ( ) Single ( ) Married ( ) Widow	EDUCATION: Middle school: ( ) Complete ( ) Incomplete High school: ( ) Complete ( ) Incomplete Higher education: ( ) Complete ( ) Incomplete			COLOR/RACE ( ) Black ( ) East Asian ( ) White ( ) Indigenous Brazilian ( ) Multiracial	
OCCUPATION: ( ) Self-employed ( ) Work for an employer ( ) Work at home			Number of children: ____________		
What is the approximate family income? ( ) Up to 1 minimum wage ( ) 2 to 3 minimum wages ( ) More than 3 minimum wages					
**PART II – DATA ON CURRENT PREGNANCY AND BREASTFEEDING**					
Type of delivery: ( ) Cesarean ( ) Natural		Did you have prenatal care? ( ) Yes ( ) No No. of consultations ( ) 1 to 3 ( ) 4 to 6 ( ) 7 to 8 ( ) + than 8			
Sex: ( ) Female ( ) Male		Date of birth:			
Do you currently have any complaint? ( ) cracked nipple ( ) Sore nipple ( ) Breastfeeding pain ( ) Others: _________					
Is the baby on exclusive breastfeeding? ( ) Yes ( ) No If not, what type of feeding is the baby receiving? ( ) mixed breastfeeding ( ) Infant formula					

Source: The authors

## Appendix 2

**Table:** Structured questionnaire applied six months after birth

	DATE:
NAME OF THE MOTHER:	
NAME OF THE BABY:	
Was the baby on exclusive breastfeeding until the 6^th^ month? ( ) Yes ( ) No Is the baby currently breastfeeding? ( ) Yes ( ) No If so, what type of breastfeeding is the baby having? ( ) exclusive breastfeeding (EBF) ( ) supplemented breastfeeding (SBF) ( ) mixed or partial breastfeeding (MBF) ( ) infant formula (IF)	
Have you introduced baby food? ( ) Yes ( ) No	
If so, when did you introduce baby food? __________	

Source: The authors
