# Supplementary material for: Factors related to early weaning in babies born at term in a public maternity
Source: Codas. 2024 Aug 5;36(5):e20240030. doi: 10.1590/2317-1782/20242024030en (PMC11340870; doi:10.1590/2317-1782/20242024030en)
Supplement: Translation

# Translation: Fatores relacionados ao desmame precoce em bebês nascidos a termo em uma maternidade pública

**DOI:** 10.1590/2317-1782/20242024030pt

**Authors:** Sandra Raquel de Melo Gomes, Mirelly Sabrina Santos Silva, Andréa Rodrigues Motta, Estevam Barbosa de Las Casas, Renata Maria Moreira Moraes Furlan

**Affiliations:** 1 Programa de Pós-graduação em Ciências Fonoaudiológicas, Faculdade de Medicina, Universidade Federal de Minas Gerais – UFMG - Belo Horizonte (MG), Brasil.; 2 Residência Multiprofissional, Hospital Metropolitano Odilon Behrens - Belo Horizonte (MG), Brasil.; 3 Departamento de Fonoaudiologia, Faculdade de Medicina, Universidade Federal de Minas Gerais – UFMG - Belo Horizonte (MG), Brasil.; 4 Departamento de Engenharia de Estruturas, Escola de Engenharia, Universidade Federal de Minas Gerais – UFMG - Belo Horizonte (MG), Brasil.

**Keywords:** Aleitamento Materno, Desmame, Nutrição da Criança, Fatores Sociodemográficos, Saúde Materno-infantil

## Abstract

**Objetivo:**

analisar como os fatores socioeconômicos, da gestação e do parto se relacionam com a situação da alimentação no sexto mês de vida de bebês nascidos a termo.

**Método:**

estudo observacional longitudinal, com 98 mães de bebês termos. A coleta de dados foi estruturada pela captação das informações referentes à história clínica e ao momento do parto nos prontuários dos bebês, seguida da aplicação de dois questionários, com questões referentes a dados sociodemográficos, dados pré e pós-gestacionais e da alimentação do bebê, sendo o primeiro respondido durante a internação hospitalar e o segundo, por contato telefônico, no 6° mês de vida. Foi realizada análise descritiva dos dados, por meio da distribuição de frequência das variáveis categóricas, análise inferencial utilizando o teste Qui-quadrado de Pearson e análise multivariada por regressão logística binária, adotando-se, para inclusão no modelo final, o nível de significância de 5%.

**Resultados:**

houve associação entre aleitamento materno exclusivo no 6º mês e escolaridade materna e entre o início da introdução alimentar e a renda familiar. Mães com ensino superior apresentaram 4,82 vezes mais chances de amamentarem os filhos de forma exclusiva até o sexto mês. Famílias de menor renda (até um salário mínimo) tiveram 2,54 vezes mais chances de iniciarem a introdução alimentar antes do sexto mês do que as famílias de maior renda.

**Conclusão:**

maior escolaridade materna foi fator preditor para o aleitamento materno exclusivo ao 6º mês e maior renda familiar foi fator preditor para introdução alimentar após o 6º mês.

## INTRODUÇÃO

O leite materno é o alimento mais apropriado para nutrição do bebê. A Organização Mundial da Saúde (OMS)^(1)^ e o Ministério da Saúde (MS) do Brasil^(2)^ recomendam que o recém-nascido receba o leite materno ainda na primeira hora de vida e o mantenha de forma exclusiva até o sexto mês. Após este período, orientam a manutenção do aleitamento materno de forma complementada com outros alimentos, até, pelo menos, 2 anos de idade.

O Estudo Nacional de Alimentação e Nutrição Infantil realizado em 2019^(3)^, ao investigar a prevalência e práticas de aleitamento materno (AM) em crianças brasileiras menores de dois anos, apontou que a duração média tem sido abaixo da recomendação de seis meses para o aleitamento materno exclusivo (AME) e de dois anos ou mais para o aleitamento complementado. O AM é praticado, em média, por 15,9 meses e o AME é realizado, em média, por apenas três meses, sendo que apenas 45,8% das crianças até seis meses de vida recebem AME. Este cenário está distante da meta estabelecida pela OMS: pelo menos, 70% das crianças com menos de seis meses de vida em AME até 2030^(3)^.

Com relação aos benefícios para a saúde do bebê, o aleitamento materno é fonte de nutrientes e anticorpos, contribui para a redução da mortalidade infantil, fortalece vínculo entre mãe e filho, estimula o desenvolvimento motor oral, contribui para o desenvolvimento das microbiotas digestiva, cutânea e respiratória, além de ter influência positiva na saúde da criança por toda a vida^(2,4)^. Quanto à saúde da mulher, o ato de amamentar previne câncer de mama, ovário e útero, reduz o risco do desenvolvimento de diabetes tipo 2 e contribui para a sua saúde mental^(2)^.

Apesar de todos estes benefícios, a decisão por amamentar não é algo imediato e simples; dependendo do contexto que a mulher estiver inserida, a amamentação pode ser um momento de dificuldades e inseguranças, sendo influenciada por cargas culturais e emocionais. No período da gestação e pós-parto, a mulher é exposta a muitas opiniões, crenças e relatos de vivências das pessoas do seu ciclo de convivência, que podem estimular ou não a amamentação^(5)^. As crenças e mitos relacionados à amamentação e ao leite materno, bem como o surgimento de dores e traumas mamilares - muitas vezes consequentes da falta de experiência materna, associados à falta de apoio e orientação de profissionais da saúde, podem ser motivadores do desmame precoce^(5)^.

Diante destas dificuldades, o leite materno acaba sendo complementado ou substituído por fórmulas infantis e, até mesmo, por outros alimentos, o que pode resultar no desmame do bebê^(6)^. A oferta da fórmula com mamadeira pode alterar o padrão de sucção do bebê, resultando em maior dificuldade de sucção no seio materno e, por sua vez, levar à recusa deste e consequentemente a uma redução na produção do leite materno por falta de estímulo^(7)^. A oferta de outros alimentos de forma precoce expõe a criança ao risco de cólicas e/ou diarreia, visto que o organismo do bebê ainda não está preparado para processar essas substâncias antes dos seis meses^(7)^. Na presença de outros alimentos, o número de mamadas tende a reduzir, o que também resultará no menor estímulo e menor produção do leite materno^(7)^.

Desta maneira, sabendo os benefícios que o leite materno traz, é importante conhecer os fatores que influenciam na continuidade do AM, para que seja possível entender e criar maneiras de apoiar as mães neste processo, evitando os prejuízos relacionados ao desmame precoce. Nesse sentido, o objetivo deste estudo foi analisar como os fatores socioeconômicos, da gestação e do parto se relacionam com a situação da alimentação no sexto mês de vida de bebês nascidos a termo.

## MÉTODO

Trata-se de um estudo observacional longitudinal. A amostra foi obtida de forma aleatória não randomizada, sendo constituída por mães de bebês termos, internados no setor Alojamento Conjunto do Hospital Odilon Behrens. O estudo foi aprovado pelo Comitê de Ética em Pesquisa da instituição sob o parecer número 4.480.984. Todas as participantes assinaram o Termo de Consentimento Livre e Esclarecido (TCLE).

Foram considerados critérios de inclusão: idade superior a 18 anos, o filho ser recém-nascido termo (idade gestacional maior ou igual a 37 semanas) e estar amamentando o filho internado na unidade Alojamento Conjunto.

Foram considerados como critérios de exclusão: mães cujos recém-nascidos apresentaram doenças congênitas cardíacas ou pulmonares severas e síndromes genéticas ou alterações estruturais orofaciais. Estas condições clínicas podem interferir na amamentação, pois condicionam o recém-nascido a apresentar maiores riscos de incoordenação durante as funções de sucção, deglutição e respiração. Também foram excluídas as mães portadoras de doenças ou procedimentos terapêuticos que apresentassem contraindicação para o aleitamento materno ou que pudessem ter alguma interferência neste. Ainda, foram excluídas mães que não responderam o segundo questionário da pesquisa.

A coleta de dados foi estruturada pela captação das informações referentes à história clínica e ao momento do parto - consultadas nos prontuários dos bebês, seguida da aplicação de dois questionários às participantes, sendo um durante a internação hospitalar e outro seis meses após (Apêndices 1 e 2).

O primeiro questionário, constituído de duas partes, foi aplicado durante a internação hospitalar, no pós-parto, nos meses de janeiro e fevereiro de 2020. A primeira parte deste questionário correspondia aos dados sociodemográficos (idade materna, cor/raça, estado civil, escolaridade, profissão e renda familiar) e aos dados das gestações anteriores (número de filhos) e a segunda parte deste questionário investigava os dados referentes à gestação atual e aleitamento materno (número de consultas de pré-natal, sexo do bebê, tipo de parto, aleitamento exclusivo na alta hospitalar e queixas sobre amamentação).

O segundo questionário foi aplicado seis meses após o nascimento da criança, por contato telefônico. O questionário foi constituído pelas seguintes perguntas sobre a situação da alimentação atual do bebê: “esteve em aleitamento exclusivo até o 6° mês?” – opções de resposta: sim ou não; “tipo de aleitamento no 6° mês?” – opções de resposta: aleitamento materno exclusivo (AME), aleitamento materno complementado (AMC), aleitamento materno misto ou parcial (AMM) e aleitamento artificial (AA) (Quadro 1)^(8)^; “está em aleitamento materno?” opções de respostas: sim ou não; “iniciou a oferta de papa?” – opções de resposta: sim ou não; e “período de início da papa”- as participantes respondiam esta pergunta de forma livre, sendo que, para análise estatística dos dados, as pesquisadoras optaram por categorizar as respostas em “até o 4° mês" e “a partir do 5° mês”. Para esta variável ainda foi considerada a categoria “não se aplica” – para bebês que, no momento do contato telefônico, ainda não tinham iniciado a introdução alimentar.

**Quadro 1:** Classificação dos tipos de aleitamento

Tipo de aleitamento	Definição
Aleitamento materno exclusivo	Quando a criança recebia apenas leite materno.
Aleitamento materno complementado	Quando a criança recebia, além do leite materno, qualquer alimento sólido ou semissólido com a finalidade de complementá-lo, e não de substituí-lo.
Aleitamento materno misto ou parcial	Quando a criança recebia leite materno e outros tipos de leite.
Aleitamento artificial	Quando a criança recebia apenas outro tipo de leite que não o materno.

Fonte: Brasil^(8)^.

Os dados dos dois questionários foram registrados em uma planilha do Microsoft Excel e, posteriormente, submetidos à análise estatística inferencial. Configuraram-se como variáveis resposta desta pesquisa a presença de aleitamento materno exclusivo até o sexto mês, a presença de algum tipo de aleitamento ao sexto mês, o tipo de aleitamento que estava sendo realizado no sexto mês e a idade de início da introdução alimentar. Como variáveis explicativas foram avaliadas: idade materna, estado civil, escolaridade, cor/raça, profissão, renda familiar, número de filhos, tipo de parto, realização do pré-natal, sexo do bebê, ocorrência de AME na alta hospitalar e queixas sobre a amamentação.

Foi realizada análise descritiva dos dados, por meio da distribuição de frequência das variáveis categóricas. Para as análises de associação, foi utilizado o teste Qui-quadrado de Pearson, sendo consideradas como associações estatisticamente significantes as que apresentaram valor de p≤0,05. Foi realizada ainda, uma análise par a par, utilizando o teste Qui-quadrado de Pearson, entre as categorias da escolaridade materna e o aleitamento materno exclusivo até o 6° mês, em que foram consideradas como associações estatisticamente significantes as que apresentaram valor de p≤0,05. Para entrada, processamento e análise dos dados foi utilizado o software SPSS, versão 25.0.

Foi realizada, ainda, análise multivariada por regressão logística binária. As variáveis explicativas, selecionadas para o modelo, foram aquelas que apresentaram associação, nas análises bivariadas, com presença de AM ao sexto mês, presença de AME ao sexto mês e início da introdução alimentar antes do sexto mês, adotando-se o nível de significância de até 20% (p=<0,20). Para inclusão no modelo final, adotou-se o nível de significância de 5%. A magnitude das associações foi avaliada pelas razões de chances (*Odds Ratio*) e seus respectivos intervalos de confiança de 95%.

## RESULTADOS

Na primeira etapa, 224 participantes responderam ao questionário. Na segunda etapa, 126 participantes não foram localizadas, sendo excluídas do estudo. Dessa forma, 98 participantes compuseram a amostra da presente pesquisa.

A maioria das mães que participaram do estudo tinha idade entre 21 e 35 anos (75,5%). Quanto ao estado civil, 64 mães eram solteiras (65,3%). No que diz respeito à escolaridade, a maioria tinha ensino médio completo (73,5%) e o restante dividiu-se igualmente entre ensino fundamental e ensino superior. Quanto à classificação de cor/raça, a maior parte das participantes classificou-se como parda (63,3%), seguida da classificação preta, branca e, por fim, amarela, com o menor percentual. Grande parte das mães trabalhava como autônoma ou em casa e relatou renda familiar de dois ou mais salários mínimos (55,1%).

Em relação às consultas de pré-natal, a maioria (83,7%) das participantes relatou ter realizado seis ou mais consultas, a maior parte (55,1%) teve parto normal e era multípara (53,1%). No que diz respeito aos bebês, 56,1% eram do sexo masculino. A maioria (89,8%) deles encontrava-se em AME no momento da alta hospitalar pós-parto.

Quanto aos dados relativos ao aleitamento materno, foi observado que 60,2% das participantes apresentaram queixas, sendo as mais frequentes fissuras e escoriações mamárias e dor ao amamentar. A maior parte dos bebês (83,7%) não recebeu AME até o 6° mês de vida. Quanto à presença do aleitamento materno no sexto mês, 64,3% dos bebês não tinham contato com o leite materno. O tipo de aleitamento predominante no 6° mês de vida foi o aleitamento artificial (34,7%), seguido do misto (31,6%), complementado (17,3%) e, por fim, do aleitamento materno exclusivo (16,3%). Em relação à introdução alimentar, a maioria dos bebês iniciou a partir do 5° mês (41,8%), 17,3% deles iniciaram com 4 meses ou antes e 40,8% não haviam iniciado.

Ao realizar análise de associação entre estar em aleitamento materno exclusivo até o 6º mês de vida com os dados sociodemográficos, dados das gestações anteriores e da gestação atual (Tabela 1), foi possível observar que houve associação com significância estatística entre aleitamento materno exclusivo no 6º mês e escolaridade materna. Por meio de análise par a par entre as categorias da escolaridade, observou-se diferença estatística entre o ensino superior e o ensino médio (p=0,010). A partir desta análise, foi possível observar que mães com ensino médio mantinham menos o aleitamento materno exclusivo, quando comparadas com mães com ensino superior. Não foi identificada associação com significância estatística ao realizar análise entre estar em aleitamento materno (não exclusivo) ao sexto mês e dados sociodemográficos e pré/pós-natais, também descritos na Tabela 1.

**Tabela 1:** Análise da associação entre as variáveis aleitamento materno ao sexto mês e aleitamento materno exclusivo até o sexto mês e os dados sócio demográficos e pré/pós-natais

**Variáveis**	**Aleitamento materno exclusivo até o 6º mês**			**Aleitamento materno ao 6º mês**		
	**Sim N (%)**	**Não N (%)**	**Valor de p**	**Sim N (%)**	**Não N (%)**	**Valor de p**
**Idade da mãe**						
Até 20 anos	2 (12,5)	12 (14,6)	0,931	2 (10,5)	10 (15,9)	0,595
21 a 35 anos	12 (75,0)	62 (75,6)		16 (84,2)	46 (73,0)	
Acima de 35 anos	2 (12,5)	8 (9,8)		1 (5,3)	7 (11,1)	
**Estado civil**						
Solteira	10 (62,5)	54 (65,9)	0,797	11 (57,9)	43 (68,3)	0,404
Casada	6 (37,5)	28 (34,1)		8 (42,1)	20 (31,7)	
**Escolaridade materna**						
Fundamental ^A,B^	3 (18,8)	10 (12,2)	0,004*	2 (10,5)	8 (12,7)	0,801
Médio ^A^	7 (43,8)	65 (79,3)		16 (84,2)	49 (77,8)	
Superior ^B^	6 (37,4)	7 (8,5)		1 (5,3)	6 (9,5)	
**Cor/Raça**						
Preto	5 (31,3)	17 (20,7)	0,603	1 (5,3)	16 (25,4)	0,293
Amarelo	1 (6,2)	2 (2,4)		0 (0,0)	2 (3,2)	
Branco	2 (12,5)	9 (11,0)		2 (10,5)	7 (11,1)	
Pardo	8 (50,0)	54 (65,9)		16 (84,2)	18 (60,3)	
**Profissão**						
Autônomo ou em casa	9 (56,3)	53 (64,6)	0,525	13(68,4)	40 (63,5)	0,694
Trabalha em empresa	7 (43,7)	29 (35,4)		6 (31,6)	23 (36,5)	
**Renda familiar**						
Até um salário mínimo	6 (37,5)	38 (43,6)	0,515	10 (52,6)	28 (44,4)	0,530
2 ou mais salários mínimos	10 (62,5)	44 (53,7)		9 (47,4)	35 (55,6)	
**Primiparidade**						
Primípara	11 (68,8)	35 (42,7)	0,056	7 (36,8)	28 (44,4)	0,557
Multípara	5 (31,2)	47 (57,3)		12 (63,2)	35 (55,6)	
**Tipo de parto**						
Cesárea	10 (62,5)	34 (41,5)	0,122	10 (52,6)	24 (38,1)	0,260
Normal	6 (37,5)	48 (58,5)		9 (47,4)	39 (61,9)	
**Pré-natal**						
Até seis consultas	2 (12,5)	13 (16,0)	0,720	5 (26,3)	8 (12,9)	0,163
Seis consultas ou mais	14 (87,5)	68 (84,0)		14 (73,7)	54 (87,1)	
**Sexo do bebê**						
Feminino	7 (43,8)	36 (43,9)	0,991	9 (47,4)	27 (42,9)	0,728
Masculino	9 (53,6)	46 (56,1)		10 (52,6)	36 (57,1)	
**Aleitamento exclusivo na alta hospitalar**						
Sim	15 (93,8)	73 (89,0)	0,568	34 (97,1)	54 (85,7)	0,081
Não	1 (6,3)	9 (11,0)		1 (2,9)	9 (14,3)	
**Queixas sobre amamentação**						
Sim	9 (53,6)	50 (61,0)	0,724	15 (78,9)	35 (55,6)	0,067
Não	7 (43,8)	32 (39,0)		4 (21,1)	28 (44,4)	

Teste Qui-quadrado de Pearson

* = valor de p≤0,05

**Legenda:** N = número de indivíduos, varia devido a dados faltantes. Letras sobrescritas diferentes indicam diferença estatística entre os grupos, enquanto letras sobrescritas iguais indicam ausência de diferença estatística entre os grupos.

A análise de associação entre o tipo de aleitamento no sexto mês e dados sociodemográficos e pré/pós-natais (Tabela 2) revelou ausência de significância estatística em quaisquer das associações analisadas.

**Tabela 2:** Análise de associação entre o tipo de aleitamento e dados sociodemográficos e pré/pós-natais

**Variáveis**	**Tipo de aleitamento**					
	**AME N (%)**	**Aleit.Com. N (%)**	**Misto N (%)**	**Artificial N (%)**	**Total N (%)**	**Valor de p**
**Idade da mãe**						
Até 20 anos	2 (14,3)	1 (7,1)	6 (42,9)	5 (35,7)	14 (100,0)	0,881
21 a 35 anos	12 (16,2)	15 (20,3)	22 (29,7)	25 (33,8)	74 (100,0)	
Acima de 35 anos	2 (20,0)	1 (10,0)	3 (30,0)	4 (40,0)	10 (100,0)	
**Estado civil**						
Solteira	10 (15,6)	9 (14,1)	21 (32,8)	24 (37,5)	64 (100,0)	0,636
Casada	6 (17,6)	8 (23,5)	10 (29,4)	10 (29,4)	34 (100,0)	
**Escolaridade materna**						
Fundamental	3 (23,0)	2 (15,4)	2 (15,4)	6 (46,2)	13 (100,0)	0,056
Médio	7 (9,7)	14 (19,4)	25 (34,7)	26 (36,2)	72 (100,0)	
Superior	6 (46,2)	1 (7,7)	4 (30,8)	2 (15,3)	13 (100,0)	
**Cor/Raça**						
Preto	5 (22,7)	1 (4,5)	6 (27,3)	10 (45,5)	22 (100,0)	0,692
Amarelo	1 (33,3)	0 (0,0)	1 (33,3)	1 (33,4)	3 (100,0)	
Branco	2 (18,2)	2 (18,2)	3 (27,3)	4 (36,3)	11 (100,0)	
Pardo	8 (12,9)	14 (22,6)	21 (33,9)	19 (30,6)	62 (100,0)	
**Profissão**						
Autônomo ou em casa	9 (14,5)	12 (19,4)	17 (27,4)	24 (38,7)	62 (100,0)	0,482
Trabalha em empresa	7 (19,4)	5 (13,9)	14 (38,9)	10 (27,8)	36 (100,0)	
**Renda familiar**						
Até um salário mínimo	6 (13,6)	10 (22,7)	12 (27,3)	16 (36,4)	44 (100,0)	0,526
Dois ou mais salários mínimos	10 (18,5)	7 (13,0)	19 (35,2)	18 (33,3)	54 (100,0)	
**Primiparidade**						
Primípara	11 (23,9)	6 (13,1)	18 (39,1)	11 (23,9)	46 (100,0)	0,058
Multípara	5 (9,6)	11 (21,1)	13 (25,0)	23 (44,3)	52 (100,0)	
**Tipo de parto**						
Cesárea	10 (22,7)	9 (20,5)	11 (25,0)	14 (31,8)	44 (100,0)	0,290
Normal	6 (11,2)	8 (14,8)	20 (37,0)	20 (37,0)	54 (100,0)	
**Pré-natal**						
Até seis consultas	2 (13,3)	4 (26,7)	3 (20,0)	6 (40,0)	15 (100,0)	0,586
Seis consultas ou mais	14 (17,1)	13 (15,9)	28 (34,1)	27 (32,9)	82 (100,0)	
**Sexo do bebê**						
Feminino	7 (16,3)	8 (18,6)	11 (25,6)	17 (39,5)	43 (100,0)	0,688
Masculino	9 (16,4)	9 (16,4)	20 (36,3)	17 (30,9)	55 (100,0)	
**Aleitamento exclusivo alta hospitalar**						
Sim	15 (17,0)	17 (19,3)	27 (30,7)	29 (33,0)	88 (100,0)	0,361
Não	1 (10,0)	0 (0,0)	4 (40,0)	5 (50,0)	10 (100,0)	
**Queixas sobre amamentação**						
Sim	9 (15,3)	14 (23,7)	16 (27,0)	20 (33,3)	59 (100,0)	0,206
Não	7 (17,9)	3 (7,7)	15 (38,5)	14 (35,9)	39 (100,0)	

**Legenda:** N = número de indivíduos; AME= Aleitamento materno exclusivo; Aleit.Comp.= Aleitamento complementado.

Teste Qui-quadrado de Pearson

Na análise de associação entre o início da introdução alimentar antes do 6º mês e dados sociodemográficos e pré/pós-natais (Tabela 3), houve associação com significância estatística entre o início da introdução alimentar e a renda familiar. Observou-se que as famílias com maior renda iniciaram a introdução alimentar posteriormente, quando comparadas às famílias de menor renda. Ao se considerar as idades mais prevalentes de início da introdução alimentar (quarto e quinto mês de vida do bebê) não foi encontrada associação significativa com dados sociodemográficos e pré/pós-natais.

**Tabela 3:** Análise de associação entre início da introdução alimentar e dados sociodemográficos e pré/pós-natais

**Variáveis**	**Iniciou a introdução alimentar**			**Idade da introdução alimentar**		
	**Sim N (%)**	**Não N (%)**	**Valor de p**	**Até 4º mês N (%)**	**A partir 5º mês N (%)**	**Valor de p**
**Idade da mãe**						
Até 20 anos	8 (14,5)	4 (14,8)	0,881	3 (17,6)	6 (14,6)	0,959
21 a 35 anos	41 (74,6)	21 (77,8)		12 (70,6)	30 (73,2)	
Acima de 35 anos	6 (10,9)	2 (7,4)		2 (11,8)	5 (12,2)	
**Estado civil**						
Solteira	33 (60,0)	21 (77,8)	0,111	9 (52,9)	27 (65,9)	0,356
Casada	22 (40,0)	6 (22,2)		8 (47,1)	14 (34,1)	
**Escolaridade materna**						
Fundamental	8 (14,5)	2 (7,4)	0,608	3 (17,6)	5 (12,2)	0,709
Médio	42 (76,4)	23 (85,2)		12 (70,6)	33 (80,5)	
Superior	5 (9,1)	2 (7,4)		2 (11,8)	3 (7,3)	
**Cor/Raça**						
Preto	10 (18,2)	7 (25,9)	0,669	4 (23,5)	7 (17,1)	0,218
Amarelo	2 (3,6)	0 (0,0)		2 (11,8)	0 (0,0)	
Branco	6 (10,9)	3 (11,1)		2 (11,8)	4 (9,8)	
Pardo	37 (67,3)	17 (63,0)		9 (52,9)	30 (73,1)	
**Profissão**						
Autônomo ou em casa	38 (69,1)	15 (55,6)	0,228	11 (64,7)	29 (70,7)	0,652
Trabalha em empresa	17 (30,9)	12 (44,4)		6 (35,3)	12 (29,3)	
**Renda familiar**						
Até um salário mínimo	30 (54,5)	8 (29,6)	0,033*	11 (64,7)	20 (48,8)	0,268
2 ou mais salários mínimos	25 (45,5)	19 (70,4)		6 (35,3)	21 (51,2)	
**Primiparidade**						
Primípara	22 (40,0)	13 (48,1)	0,483	6 (35,3)	16 (39,0)	0,790
Multípara	33 (60,0)	14 (51,9)		11 (64,7)	25 (61,0)	
**Tipo de parto**						
Cesárea	25 (45,5)	9 (33,3)	0,295	7 (41,2)	18 (43,9)	0,849
Normal	30 (54,5)	18 (66,7)		10 (58,8)	23 (56,1)	
**Pré-natal**						
Até seis consultas	10 (18,2)	3 (11,5)	0,447	2 (11,8)	9 (22,0)	0,368
Seis consultas ou mais	45 (81,8)	23 (88,5)		15 (88,2)	32 (78,0)	
**Sexo do bebê**						
Feminino	25 (45,5)	9 (33,3)	0,295	4 (23,5)	19 (46,3)	0,106
Masculino	30 (54,5)	18 (66,7)		13 (76,5)	22 (53,7)	
**Aleitamento exclusivo na alta hospitalar**						
Sim	51 (92,7)	22 (81,5)	0,126	16 (94,1)	38 (92,7)	0,844
Não	4 (7,3)	5 (18,5)		1 (5,9)	3 (7,3)	
**Queixas sobre amamentação**						
Sim	35 (63,6)	15 (55,6)	0,481	11 (64,7)	26 (63,4)	0,926
Não	20 (36,4)	12 (44,4)		6 (35,3)	15 (36,6)	

Teste Qui-quadrado de Pearson

* = valor de p≤0,05

**Legenda:** N = número de indivíduos, varia devido a dados faltantes e devido aos bebês inclusos na categoria “não se aplica” (N=40) – correspondente aos bebês que no sexto mês ainda não tinham iniciado a introdução alimentar

Os resultados da análise multivariada (Tabela 4) indicam que a escolaridade pode ser considerada fator preditor para o aleitamento materno exclusivo até o sexto mês, sendo que mães com ensino superior apresentaram 4,82 vezes mais chances de amamentarem os filhos de forma exclusiva até o sexto mês. A renda familiar foi fator preditor para o início da introdução alimentar depois dos seis meses, visto que as famílias de menor renda (até um salário mínimo) tiveram 2,54 vezes mais chances de iniciarem oferta da introdução alimentar antes do sexto mês do que as famílias de maior renda.

**Tabela 4:** Modelo de regressão logística para as variáveis aleitamento materno exclusivo ao sexto mês, aleitamento materno no sexto mês e introdução alimentar antes do sexto mês

Variáveis	Razão de chances	Valor de p	Intervalo de confiança 95%	
			Mínimo	Máximo
**AME ao 6º mês**				
Escolaridade (Ensino Superior)	4,82	0,026	1,209	19,260
Primiparidade (Primípara)	1,84	0,345	0,518	6,549
Tipo de parto (Normal)	0,45	0,191	0,143	1,473
**AM ao 6º mês**				
Pré-natal (até 6 consultas)	2,56	0,154	0,704	9,291
AME na alta hospitalar (Sim)	1	-	-	-
Queixas (Sim)	2,95	0,081	0,877	9,904
**Introdução alimentar antes do 6º mês**				
Estado civil (casada)	1,85	0,177	0,757	4,513
Renda (até um salário)	2,54	0,031	1,091	5,918
AME à alta hospitalar (sim)	2,12	0,276	0,547	8,255

**Legenda:** AME = aleitamento materno exclusivo, AM = aleitamento materno

## DISCUSSÃO

No presente estudo foi encontrada associação significativa entre aleitamento materno exclusivo até o 6º mês e escolaridade materna. Notou-se que em cada categoria de escolaridade (ensino fundamental, ensino médio e ensino superior) a maioria das mães não estava em amamentação exclusiva até o 6° mês. A maior parte delas tinha ensino médio completo e não mantiveram amamentação exclusiva até o 6° mês. Entretanto, foi possível identificar, por avaliação par a par, que as mães com ensino superior amamentaram mais até o sexto mês de forma exclusiva do que as mães que possuíam ensino médio. Dessa forma, elencou-se a categoria Ensino Superior para entrada no modelo de regressão logística, confirmando a escolaridade como fator de proteção ao aleitamento materno exclusivo até o sexto mês, tendo as mães com ensino superior 4,82 vezes mais chances de amamentarem os filhos de forma exclusiva até o sexto mês.

Esses achados são similares ao que a literatura aponta. Silva et al.^(6)^, em uma revisão da literatura, verificaram que a escolaridade mais baixa é um fator relacionado ao desmame precoce e sugeriram que o fato de o grupo de mães com menor escolaridade ter menos acesso à informação explica o porquê de deixarem de amamentar precocemente. Esses dados são reafirmados por Nabate et al.^(9)^ que apontaram que mães com menor nível de escolaridade demonstram tendência significativa à prática do desmame precoce. Em concordância, Barbosa et al.^(10)^ referiram que mães com menos de oito anos de escolaridade (ensino fundamental incompleto) tendem a abandonar o AME precocemente e reforçaram que mulheres com pouca ou nenhuma instrução desconhecem a importância do AME para a saúde do bebê. Nesse sentido, a promoção e ampliação das campanhas educativas à população poderia ser útil no aumento das taxas de AME.

Outros autores^(11)^ também referem que a baixa escolaridade está associada à menor duração do aleitamento materno. Baseados em um estudo que utilizou dados da Pesquisa Estadual de Saúde e Nutrição de Pernambuco (PESN), de 1991, 1997 e 2006, os autores^(11)^ apontaram que mulheres com nove ou mais anos de escolaridade apresentaram maior prevalência de AME ao sexto mês quando comparadas àquelas com menor escolaridade, configurando, assim, a maior escolaridade como fator de proteção na duração do AME. Tais autores^(11)^ sugerem que o maior número de consultas no pré-natal pode incentivar a continuidade do aleitamento materno entre as mães com escolaridade mais baixa, visto que as consultas são um espaço de oportunidade para a realização de orientações e fortalecimento de conhecimentos sobre o processo de amamentar.

Nos resultados do presente estudo, também foi encontrada a associação estatisticamente significativa entre renda familiar e a introdução alimentar precoce. Dentre as mães que não tinham iniciado a introdução alimentar aos filhos até o 6º mês, grande parte tinha maior renda familiar (dois ou mais salários mínimos). O modelo de regressão mostrou que famílias de menor renda (até um salário mínimo) tiveram 2,54 vezes mais chances de iniciarem a introdução alimentar antes do sexto mês do que as famílias de maior renda.

Melo et al.^(12)^, em estudo realizado com pais de crianças de 0 a 2 anos de três escolas privadas de Belo Horizonte e Contagem, identificaram correlação entre os aspectos de escolaridade, ocupação fora de casa, renda familiar e ter plano de saúde com o conhecimento dos pais sobre introdução alimentar infantil. Os pais com maior conhecimento sobre introdução alimentar tinham maior escolaridade, trabalhavam fora de casa, possuíam maior renda familiar e contavam com um plano de saúde.

A literatura sugere que quanto maior o conhecimento sobre a alimentação complementar, menor é a chance de a introdução alimentar ser realizada precocemente. Além disso, a determinação da escolha sobre a composição da alimentação da criança relaciona-se diretamente com o poder de compra das famílias, o que sofre influência direta da renda familiar. A alimentação da criança abrange, portanto, aspectos socioculturais e econômicos^(12)^.

Giesta et al.^(13)^, em estudo realizado com mães de crianças de 4 a 24 meses internadas na pediatria ou emergência pediátrica de um hospital terciário na cidade de Porto Alegre, apontaram que, apesar de grande parte das mães ter recebido algum tipo de orientação sobre alimentação complementar por profissionais de saúde, foram constatadas baixa prevalência de AME e introdução alimentar inadequada. Associado a isso, observou-se alta prevalência de alimentos ultraprocessados introduzidos antes dos seis meses de vida. Estas práticas inadequadas estiveram mais presentes entre as mães de menor renda familiar, menor escolaridade, idade mais avançada e multíparas.

A caracterização da amostra do presente estudo, em que a maioria das mulheres tinha renda familiar de dois ou mais salários mínimos, era multípara e com filhos em aleitamento artificial, sugere que estas mães não iniciaram a introdução alimentar precocemente por terem condições financeiras para manter a oferta de fórmulas infantis. Quando se consegue manter a fórmula infantil, de forma atender 100% a necessidade da criança, não se identifica a necessidade de inserir outros alimentos para satisfazê-la.

Entender a relação do nível socioeconômico das famílias com o início precoce da alimentação complementar e a oferta inadequada de alimentos para esta fase viabiliza a criação de políticas de saúde que garantam práticas alimentares adequadas desde o início da alimentação e que permaneçam ao longo da infância, adolescência e vida adulta^(14)^.

A alimentação complementar foi introduzida às crianças do presente estudo, na maior parte das vezes, no 5º mês. Os demais lactentes dividiram-se nas categorias até o 4° mês e não se aplica, o que significa que a introdução alimentar ainda não havia iniciado. Os achados do estudo de Melo et al.^(12)^ concordam com os da presente pesquisa, ao demonstrarem que, na sua amostra, a introdução alimentar se deu, na maior parte das vezes, entre 0 e 5 meses, de forma precoce ao considerar o que é preconizado pelo MS e pela OMS. A introdução alimentar precoce normalmente está associada ao desmame precoce. Dessa forma, os fatores que influenciam na decisão de parar de amamentar, acabam consequentemente incentivando a oferta da alimentação complementar antes do recomendado^(7,15)^.

A introdução alimentar precoce é frequente em diversos países, seja em países desenvolvidos ou em desenvolvimento^(16-19)^. Um estudo realizado em países do Oriente Médio apontou que 78,6% das crianças do Iraque, 70% das crianças dos Emirados Árabes e 52,9% das crianças do Líbano recebem alimentação complementar entre quatro e seis meses de vida, não seguindo as recomendações da OMS^(18)^. Nesse mesmo sentido, um estudo multicêntrico com países europeus constatou que 25% das crianças avaliadas tiveram o início da alimentação complementar antes do 4° mês de vida e, ainda, que no 6° mês de vida, ao menos, 90% das crianças haviam consumido alimentos sólidos^(19)^.

Relacionado ao tipo de aleitamento no sexto mês, um ponto que chama a atenção no presente estudo é que grande parte dos bebês não estava em AME e tampouco em aleitamento materno. Dos lactentes avaliados, 34,7% estavam em aleitamento artificial e o restante dividiu-se nas outras categorias. Torquato et al.^(20)^, ao avaliarem o padrão de aleitamento materno de crianças de 0 a 24 meses de idade constataram que a maioria das crianças não estava em aleitamento materno. Ressalta-se que aquelas com idade inferior ou igual a 6 meses, em sua grande parte, não estavam recebendo leite materno nem de forma complementada, tampouco exclusivamente. Torquato et al.^(20)^ reforçam que a crença do leite materno ser insuficiente e/ou fraco é ainda muito forte e enraizada e tem grande influência na decisão das mães em ofertar outros tipos de alimentos (água, suco, outros leites e alimentos sólidos) antes dos seis meses. Outro ponto de destaque é que mais de 10% das puérperas saíram do hospital sem praticar o AME. Destas, apenas uma realizava o AME ao sexto mês. Nesse sentido, destaca-se a importância de ações destinadas a iniciação e manutenção do aleitamento materno no puerpério imediato, como, por exemplo, orientações realizadas por profissionais, aconselhamento e intervenções de apoio entre pares^(21)^.

Pinheiro et al.^(16)^, por sua vez, pontuam que muitas vezes em decorrência de dores e traumas mamilares as lactantes decidem não seguir com a amamentação, mesmo conhecendo a importância e benefícios da amamentação exclusiva até o 6º mês. Barbosa et al.^(22)^ também identificaram alta frequência de desmame precoce nos primeiros meses de vida do bebê e pontuaram como fatores associados ao fim do AME os problemas mamários, observados ainda na maternidade e que se mantiveram. Apesar de a literatura considerar as dores e traumas nas mamas como aspectos importantes para a descontinuidade do aleitamento materno e de a maior parte das mães do presente estudo terem apresentado queixas relacionadas à amamentação, a associação entre a presença da queixa e o desmame precoce não foi observada na amostra estudada.

Algumas limitações devem ser consideradas neste estudo. O número de participantes foi reduzido e isso está associado ao curto período de duração da coleta de dados. Ainda há de se considerar o cenário da coleta: hospital com características específicas - hospital metropolitano que é referência no atendimento de gravidez de alto risco, sendo assim os dados não devem ser generalizados para outras populações. Além disso, a memória materna pode ser considerada um ponto de viés do estudo, já que a aplicação do segundo questionário contou com perguntas que dependiam das lembranças maternas. Como ponto forte da presente pesquisa, tem-se o componente de acompanhamento, caracterizado por duas medidas no tempo, o que minimizou vieses que estariam presentes caso tivesse sido realizada apenas no sexto mês. Este estudo mostra-se de relevância à literatura ao elucidar como os dados socioeconômicos, da gestação e do parto se relacionam com a situação da alimentação no sexto mês de vida do bebê. Esse entendimento pode dar luz a estratégias que auxiliem a mulher no processo de amamentação, bem como na introdução alimentar, evitando o desmame precoce e suas consequências.

## CONCLUSÃO

Houve associação entre escolaridade materna e presença de AME no sexto mês – mães com ensino superior amamentam mais até o sexto mês de vida de forma exclusiva quando comparadas às mães com ensino médio. Outra associação identificada foi entre renda familiar e introdução alimentar ao sexto mês, sendo que as mães com maior renda não iniciaram a alimentação complementar antes do sexto mês.

## Apêndice 1

**Table:** Questionário estruturado para aplicação no hospital

					DATA:
**PARTE I - IDENTIFICAÇÃO E DADOS SOCIOECONÔMICOS**					
NOME:					
DATA DE NASCIMENTO:			IDADE:		
NATURALIDADE:			Nº PRONTUÁRIO:		
ENDEREÇO:			TELEFONES:		
ESTADO CIVIL: ( ) Solteira ( ) Casada ( ) Viúva	ESCOLARIDADE Fundamental: ( ) Completo ( ) Incompleto Médio: ( ) Completo ( ) Incompleto Superior: ( ) Completo ( ) Incompleto			COR/RAÇA ( ) Preto ( ) Amarelo ( ) Branco ( ) Indígena ( ) Pardo	
PROFISSÃO: ( ) autônomo ( ) Trabalho em uma empresa ( ) Trabalho em casa			Número de filhos:____________		
Qual a renda aproximada da família? ( ) Até 1 salário mínimo ( ) 2 a 3 salários mínimos ( ) Mais de 3 salários mínimos					
**PARTE II - DADOS REFERENTES À GESTAÇÃO ATUAL E ALEITAMENTO MATERNO**					
Tipo de parto: ( ) Cesárea ( ) Normal		Realizou pré-natal? ( ) Sim ( ) Não Nº de consultas ( ) 1 a 3 ( ) 4 a 6 ( ) 7 a 8 ( )+ de 8			
Sexo: ( ) Feminino ( ) Masculino		Data de Nascimento:			
Você tem alguma queixa nesse momento ? ( ) Fissuras mamárias ( ) Escoriações ( ) Dor ao amamentar ( ) Outras: _________					
O bebê está em aleitamento materno exclusivo? ( ) Sim ( ) Não Se não, qual tipo de aleitamento ele se encontra? ( ) Aleitamento misto ( ) Aleitamento artificial					

Fonte: próprios autores.

## Apêndice 2

**Table:** Questionário estruturado para aplicação seis meses após o parto

	DATA:
NOME DA MÃE:	
NOME DO BEBÊ:	
O bebê esteve em aleitamento materno exclusivo até o 6º mês? ( ) Sim ( ) Não O bebê está em aleitamento materno? ( ) Sim ( ) Não Se sim, qual tipo de aleitamento ele se encontra? ( ) aleitamento materno exclusivo (AME) ( ) aleitamento materno complementado (AMC) ( ) aleitamento materno misto ou parcial (AMM) ( ) aleitamento artificial (AA)	
Iniciou a oferta de papinhas? ( ) Sim ( ) Não	
Sem sim, indique o período de início da oferta da papa:___________	

Fonte: próprios autores.
